# Association between ambient air pollution and outpatient visits for primary acquired lacrimal duct obstruction in Hangzhou, China

**DOI:** 10.3389/fpubh.2025.1632109

**Published:** 2025-09-29

**Authors:** Qi Miao, Yuwei Wang, Peifang Xu, Xin Shi, Yihua Wu, Juan Ye, Han Wu

**Affiliations:** ^1^Eye Center, Second Affiliated Hospital of Zhejiang University School of Medicine, Hangzhou, Zhejiang Province, China; ^2^Department of Toxicology of School of Public Health and Department of Gynecologic Oncology of Women's Hospital, Zhejiang University School of Medicine, Hangzhou, Zhejiang Province, China

**Keywords:** air pollution, air pollutant, primary acquired lacrimal duct obstruction, case-crossover study, public health

## Abstract

**Background:**

Primary acquired lacrimal duct obstruction (PALDO) is the most common lacrimal drainage disease in clinics, which can be caused by multiple factors. However, few studies have investigated environmental risk factors contributing to PALDO exacerbation. This study aimed to investigate the potential association between short-term exposure to major ambient air pollutants and outpatient visits for PALDO.

**Methods:**

Data of outpatients with PALDO who visited the Eye Center of the Second Affiliated Hospital, Zhejiang University School of Medicine (Hangzhou, Zhejiang Province, China) from January 1, 2014 to December 31, 2022 were collected. The concentrations of particulate matter (PM_10_ and PM_2.5_), nitrogen dioxide (NO_2_), sulfur dioxide (SO_2_), carbon monoxide (CO) and ozone (O_3_), as well as the meteorological factors during the same period were obtained from Resource and Environment Science and Data Center, Chinese Academy of Science. A conditional logistic regression with a time-stratified case-crossover design was conducted to analyze the association between air pollutants and outpatient visits for PALDO.

**Results:**

In the single-pollutant model, significant associations were observed between PM_10_ (Odds ratio (OR) = 1.0022; 95% confidence interval (CI):1.0008, 1.004), PM_2.5_ (OR = 1.0025; 95% CI: 1.0004, 1.005), NO_2_ (OR = 1.006; 95% CI: 1.0025, 1.010), SO_2_ (OR = 1.0124; 95% CI: 1.0027, 1.022) and CO (OR = 1.3273; 95% CI: 1.0183, 1.73) and outpatient visits for PALDO. These associations remained significant after adjusting for the certain pollutant in the multi-pollutant model except NO_2_. Moreover, variations occurred between sexes, among different age groups and different seasons.

**Conclusions:**

Our study provided new and robust evidence that short-term exposure to air pollution may increase the risk of PALDO. Further studies are needed to decipher the underlying mechanisms.

## Introduction

Air pollution is a leading environmental health risk worldwide and has been associated with numerous human diseases, such as ischemic heart disease, stroke, lung cancer and chronic obstructive pulmonary disease (COPD) (1–3). According to the report of the Lancet Commission on pollution and health, air pollution can cause an estimated 9 million premature deaths each year (4). In addition, nearly 99% of the global population still exposed to unhealthy air that had failed to meet the World Health Organization (WHO) air quality guidelines (5). As the largest developing country in the world, China is experiencing remarkable social and economic growth, which also has come at a significant cost to the environment (6–9). Although the government has implemented various measures such as stricter environmental policies, technological innovation, and public health initiatives, air pollution remains a prominent public health issue right now (10–15). In recent years, increasing studies have focused on the adverse impacts of air pollution on ocular health. The morbidities of ocular diseases, such as dry eye disease, conjunctivitis, pterygium and age-related macular degeneration, have been attributed to the exposure of air pollution (16–19). However, whether air pollution could affect the lacrimal drainage diseases remains unclear.

Lacrimal drainage system (LDS) is the excretory component of the lacrimal system, providing a route for tears from the ocular surface to the nasal cavity. Besides, LDS regulates tear dynamics and contributes to tear film homeostasis, which is crucial for ocular surface health (20, 21). Anatomically, LDS consists of punctum, canaliculus, common canaliculus, lacrimal sac and nasolacrimal duct, lined by non-keratinized stratified squamous or columnar epithelium. Stenosis or blockage within any of these components would induce symptoms of epiphora and secretions, thereby resulting in lacrimal duct obstruction diseases. The patients with lacrimal duct obstruction may also suffer from one or more complications, such as conjunctivitis, keratitis, dacryocystitis and orbital cellulitis (22). Amongst the overall lacrimal drainage diseases, primary acquired lacrimal duct obstruction (PALDO) was the most common (23). In the United States, the prevalence of symptomatic acquired lacrimal duct obstruction increased and was estimated to be 30.5 cases per 100,000 persons during the period 1976–2000 (24). In fact, the etiopathogenesis of PALDO is believed to be multifactorial and has not yet been fully elucidated. To date, several studies have suggested risk factors that are implicated in PALDO, including narrowed bony nasolacrimal duct, local hormonal imbalance, nasal abnormalities, autonomic dysregulation and gastroesophageal reflux (25). However, few studies have concerned environmental risk factors.

The formation of PALDO is a complicated process, involving multiple pathological changes and both genetic and environmental risk factors. Due to the position of LDS, the luminal mucosa is constantly exposed to various environmental agents like environmental allergens, chemical contaminants and pathogenic organisms, which could induce acute or chronic inflammation within ocular surface and airways (26, 27). Although the precise pathogenesis of PALDO is unclear, evidence has shown that active inflammatory infiltrate is the common pathological character in every stage of PALDO (28). The similar pathological changes were also found in the adjacent nasal mucosa of patients with PALDO (29). Besides, the pro-inflammatory cytokines of tears were significantly elevated in PALDO patients compared to healthy controls (30). These studies have indicated that the lacrimal mucosa could also be involved in a similar inflammatory process caused by the environmental harmful factors, consequently contributing to the development of PALDO. As the well-established environmental hazards, air pollutants especially fine particulate matter < 2.5 μm in diameter (PM_2.5_) could deposit and elicit inflammatory response both in human corneal and bronchial epithelial cells (31, 32). Moreover, epidemiological studies have shown that increases in air pollutants are associated with outpatient visits for several ocular surface and respiratory diseases (33, 34). Therefore, it is valid to speculate that air pollutants might be detrimental to LDS and play an important role in the pathological mechanism of PALDO. However, to the best of our knowledge, no epidemiological study has been conducted to examine the relationship between short-term exposure to air pollution and risk of PALDO outpatient visits.

In the present study, we performed a time-stratified case-crossover study to investigate the effects of major ambient air pollutants, including particulate matter < 10 μm in diameter (PM_10_), PM_2.5_, nitrogen dioxide (NO_2_), sulfur dioxide (SO_2_), carbon monoxide (CO) and ozone (O_3_), as well as the meteorological factors, on outpatient visits for PALDO in Hangzhou, China.

## Materials and methods

### Outpatient information

Daily individual cases of PALDO during the period between January 1, 2014 and December 31, 2022 were obtained from the Eye Center of the Second Affiliated Hospital, Zhejiang University School of Medicine (Hangzhou, Zhejiang Province, China), which is the biggest ophthalmology clinic in Zhejiang Province, providing eye care service for patients from all districts of Hangzhou and the surrounding area. The definition for PALDO included H04.502-H04.506 according to the International Classification of Diseases, 10th Revision (ICD-10). H04.501 was not included because it represents congenital nasolacrimal duct obstruction. The data was obtained and comprised each patient's unique number, date of first confirmation of diagnosis, age, sex and residential address. This study adhered to the tenets of the Declaration of Helsinki and approved by the ethics committee of the Second Affiliated Hospital, Medical College of Zhejiang University.

### Air pollutants and meteorological data

The data of six ambient air pollutants (PM_10_, PM_2.5_, NO_2_, SO_2_, CO, and O_3_) from January 1, 2014 to December 31, 2022 were obtained from the Department of Ecology and Environment of Zhejiang Province. Briefly, the 24-h mean values for PM_10_, PM_2.5_, SO_2_, NO_2_, CO and the maximum 8-h value for O_3_ were collected from 12 fixed air-quality monitoring stations in 10 districts of Hangzhou: Yunxi, Shifudalou, Zhenerzhong, Xixi, Zhejiangnongda, Wolongqiao, Xiaofangdadui, Hemuxiaoxue, Binjiang, Xiasha, Linpingzhen and Chengxiangzhen. The meteorological data (daily mean temperature, relative humidity and atmospheric pressure) during the study period were obtained from Resource and Environment Science and Data Center, Chinese Academy of Science.

### Statistical analysis

A time-stratified case-crossover design was used in this study to explore the association between air pollutants and outpatient visits for PALDO. The case-crossover design, a modified version of the traditional case-control design, has been widely used to investigate transient effects of environmental exposures on health. By design, each patient serves as his/her own control, thereby reducing potential bias from time-invariant characteristics such as sex and genotype. Moreover, slowly changing confounders like smoking and drinking can be controlled by time-stratified method, since other days of the week, month and year matched to the day of the hospital visit for PALDO are selected as control days. As controls are chosen both before and after the case, each case matches to 3–4 controls depend on the number of days of the week in a particular month.

The descriptive statistics included the description of PALDO cases, air pollutants and meteorological factors, and correlation analyses were also carried out. The correlations between air pollutants and meteorological factors were analyzed by Spearman's correlation.

The association between PALDO cases and air pollution was estimated with the odds ratios (ORs) and their 95% confidence intervals (CI), which were calculated using conditional logistic regression by comparing the concentrations of air pollutants on the case and the matched control days. The conditional logistic regression analysis was performed by using a Cox proportional hazards regression model, which is a valid and efficient approach for matched case-control studies (including case-crossover designs), leveraging the mathematical equivalence between the two methods under specific conditions (35). The odds ratios were calculated with weights equal to the number of daily PALDO outpatient visits after case controls were selected and matched. As meteorological factors may be associated with PALDO, the daily mean values of each meteorological factor and air pollutant were viewed as covariates in the following formula to adjust for possible confounding by these variables:


$$\begin{array}{l} \text{ln}\left(\text{h}\left(\text{t},\text{X}\right)\right)=\text{ln}\left({\text{h}}_{0\text{i}}\left(\text{t}\right)\right)+\text{T}\beta \text{1}+\text{RH}\beta \text{2}+\text{P}\beta \text{3}+\text{C}\left(\text{P}{{\text{M}}_{\text{1}}}_{0}\right)\beta \text{4}\\ \;+\text{C}\left(\text{P}{{\text{M}}_{\text{2}.}}_{\text{5}}\right)\beta \text{5}+\text{C}\left(\text{N}{\text{O}}_{\text{2}}\right)\beta \text{6}+\text{C}\left(\text{S}{\text{O}}_{\text{2}}\right)\beta \text{7}\\ \;+\text{C}\left(\text{CO}\right)\beta \text{8}+\text{C}\left({\text{O}}_{\text{3}}\right)\;\beta \text{9} \end{array}$$

where *t* refers to the day; *X* refers to the outpatient visit; *h*(*t, X*) refers to risk function; *h*_0i_(*t*) refers to the baseline risk function; T, RH, P, C(PM_10_), C(PM_2.5_), C(NO_2_), C(SO_2_), C(CO) and C(O_3_) refer to the daily mean temperature, relative humidity, atmospheric pressure and corresponding concentrations of air pollutants, respectively; and β refers to the coefficient for each covariate. In the single-pollutant model, the acute effects of each air pollutant were evaluated with both a single lag model (lag 0 to lag 7) and a moving average lag model (lag 0–1 to lag 0–7) up to 7 days before the outpatient visits. In lag models, the best lag time was selected according to the maximum OR and the minimum *p*-values. Moreover, considering the possible interactions between air pollutants, the multi-pollutant model was applied, wherein the concentrations of copollutants in the best lags were sequentially used as variables.

The subgroup analyses were performed by sex, age and season. Specifically, patients were divided into two gender groups: male and female, and three age groups: < 40, 40–60, and >60. The whole year was divided into four seasons: spring (March to May), summer (June to August), fall (September to November) and winter (December to February), according to the seasonal characteristics of Hangzhou.

All the data management and descriptive statistics were conducted using IBM SPSS Statistics for Windows, Version 21.0 (IBM Corp., Armonk, NY, USA). The acute effects of air pollution were performed by survival package of R 3.5.2 software. All reported *p*-values were based on two-sided tests and a value of *P* < 0.05 was considered statistically significant.

## Results

A total of 2,523 outpatients from 10 districts of Hangzhou visited our eye center with the diagnosis of PALDO from January 1, 2014 to December 31, 2022. Among these patients, females (74%) and those between the ages of 40 to 60 years (48.4%) were predominant. More PALDO cases were diagnosed in the spring (30.6%) (Table 1).

**Table 1:** Characteristics of outpatient for PALDO in Eye Center of the Second Affiliated Hospital, Zhejiang University School of Medicine (2014–2022).

**Characteristics**	**Number**	**Percentage (%)**
All	2,523	100
**Sex**		
Female	1,867	74
Male	656	26
**Age (years)**		
< 40	561	22.2
40–60	1,222	48.4
>60	740	29.3
**Season**		
Spring	771	30.6
Summer	488	19.3
Fall	570	22.6
Winter	694	27.5

Summary statistics of the ambient air pollutants (PM_10_, PM_2.5_, NO_2_, SO_2_, CO, and O_3_) and meteorological factors (temperature, relative humidity and atmospheric pressure) are shown in Table 2. The mean difference between concentrations of air pollutants measured on the case days and the average over control days was 2.93 ± 33.45 μg/m^3^ for PM_10_, 1.30 ± 22.45 μg/m^3^ for PM_2.5_, −0.16 ± 12.96 μg/m^3^ for NO_2_, 0.18 ± 5.44 μg/m^3^ for SO_2_, 0.01 ± 0.18 μg/m^3^ for CO, and 0.36 ± 37.72 μg/m^3^ for O_3_. Then the correlations between air pollutants and meteorological factors were analyzed. As shown in Supplementary Table S1, the daily concentrations of air pollutants were strongly correlated with each other (*r* = 0.49–0.94) except O_3_ (*r* = −0.27 to −0.02), and weakly or moderately correlated with meteorological factors (*r* = −0.46 to 0.61).

**Table 2:** Descriptive statistics of air pollutants, the absolute difference of air pollutants between case days and the average levels over control days, and meteorological factors in the case-crossover analysis in Hangzhou, 2014–2022.

**Variable type**	**Variable name**	**Mean ±SD**	**Percentile**					**IQR**
			**0**	**25**	**50**	**75**	**100**	
Air pollution exposure in μg/m^3^ on case days (*N =* 2,523)	PM_10_	47.06 ± 29.91	7.00	45.00	68.00	101.00	305.00	56.00
	PM_2.5_	77.05 ± 42.87	4.00	26.00	39.00	61.00	233.00	35.00
	NO_2_	44.43 ± 17.53	6.00	31.00	42.00	54.00	119.00	23.00
	SO_2_	12.78 ± 8.43	3.00	7.00	10.00	16.00	78.00	9.00
	CO	0.84 ± 0.24	0.40	0.70	0.80	1.00	2.00	0.30
	O_3_	91.63 ± 49.49	4.00	55.00	82.00	125.50	275.00	70.50
Air pollution exposure difference in μg/m^3^ between case days (*N =* 2,523) and average over control days (*N =* 8,511)	PM_10_	2.93 ± 33.45	−136.00	−15.66	2.93	16.00	192.00	31.66
	PM_2.5_	1.30 ± 22.45	−101.00	−10.25	−1.00	11.00	133.75	21.25
	NO_2_	−0.16 ± 12.96	−40.33	−8.33	−1.33	6.66	73.00	14.99
	SO_2_	0.18 ± 5.44	−28.00	−1.75	0.00	1.66	42.66	3.41
	CO	0.01 ± 0.18	−0.70	−0.10	0.00	0.10	1.00	0.20
	O_3_	0.36 ± 37.72	−163.66	−21.66	−0.66	22.66	152.25	44.32
Meteorological factors in Hangzhou (2014–2022)	Mean temperature (°C)	18.19 ± 8.70	−5.00	10.60	19.00	25.30	35.60	14.70
	Relative humidity (%)	73.34 ± 14.06	27.00	64.00	74.00	84.00	98.80	20.00
	Mean pressure (hPa)	1011.3 ± 9.05	979.10	1003.50	1011.50	1018.50	1036.60	15.00

PM_10_, particulate matter < 10 μm in diameter; PM_2.5_, fine particulate matter < 2.5 μm in diameter; NO_2_, nitrogen dioxide; SO_2_, sulfur dioxide; CO, carbon monoxide; O_3_, ozone; IQR, interquartile range; SD, standard deviation.

**Figure 1:**
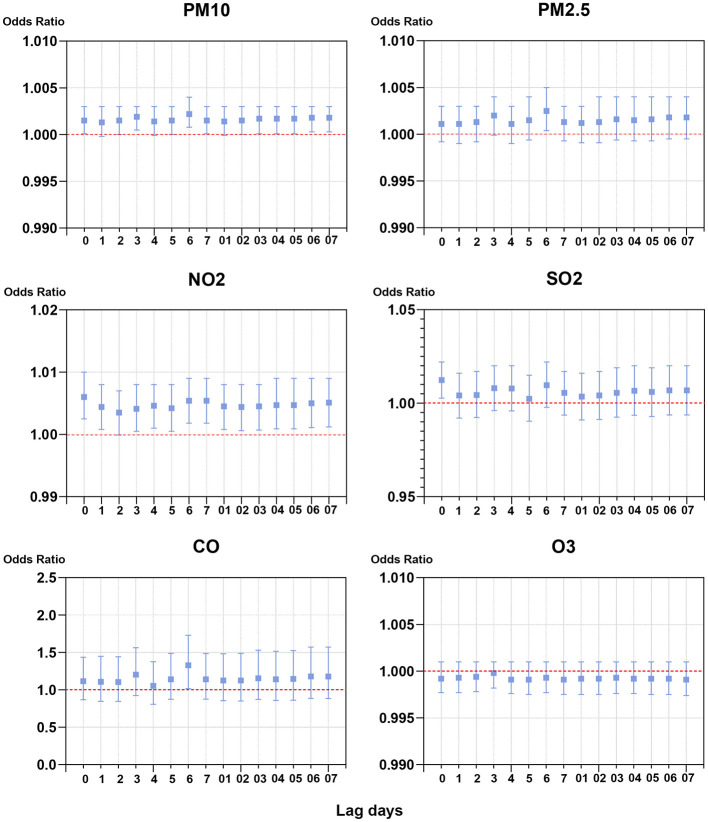
Associations of short-term exposure to air pollutants with outpatient visits for PALDO: single-pollutant model. Conditional logistic regression was performed using a Cox proportional hazards regression model to calculate the odds ratios (OR) and 95% confidence interval (CI). Each panel shows the association between PM_10_, PM_2.5_, NO_2_, SO_2_, CO, and O_3_ and PALDO cases with both a single lag model (lag 0 to lag 5) and a moving average lag model (lag 0–1 to lag 0–5). PM_10_, particulate matter <10 μm in diameter; PM_2.5_, fine particulate matter <2.5 μm in diameter; SO_2_, sulfur dioxide; NO_2_, nitrogen dioxide; CO, carbon monoxide; O_3_, ozone.

To investigate the association between exposure to single air pollutant and risk of outpatient visits for PALDO, the single-pollutant model was applied. As shown in Figure 1 and Supplementary Table S2, the best lag times were lag 6 days for PM_10_, PM_2.5_ and CO, lag 0 days for NO_2_ and SO_2_, and lag 3 days for O_3_. In the single lag model, significant positive associations between air pollutants and outpatient visits for PALDO were observed with PM_10_ lags of 0, 2, 3, 5, 6, 7 days, with PM_2.5_ lag of 6 days, with NO_2_ lags of 0, 1, 3, 4, 5, 6, 7 days, with SO_2_ lag of 0 days, and with CO lag of 6 days. Positive associations were also observed in the moving average lag model from lag 0–3 to lag 0–7 for PM_10_, and from lag 0–1 to lag 0–7 for NO_2_. In our single-pollutant model, no evidence indicated the significant association between PALDO outpatient visits and O_3_.

**Figure 2:**
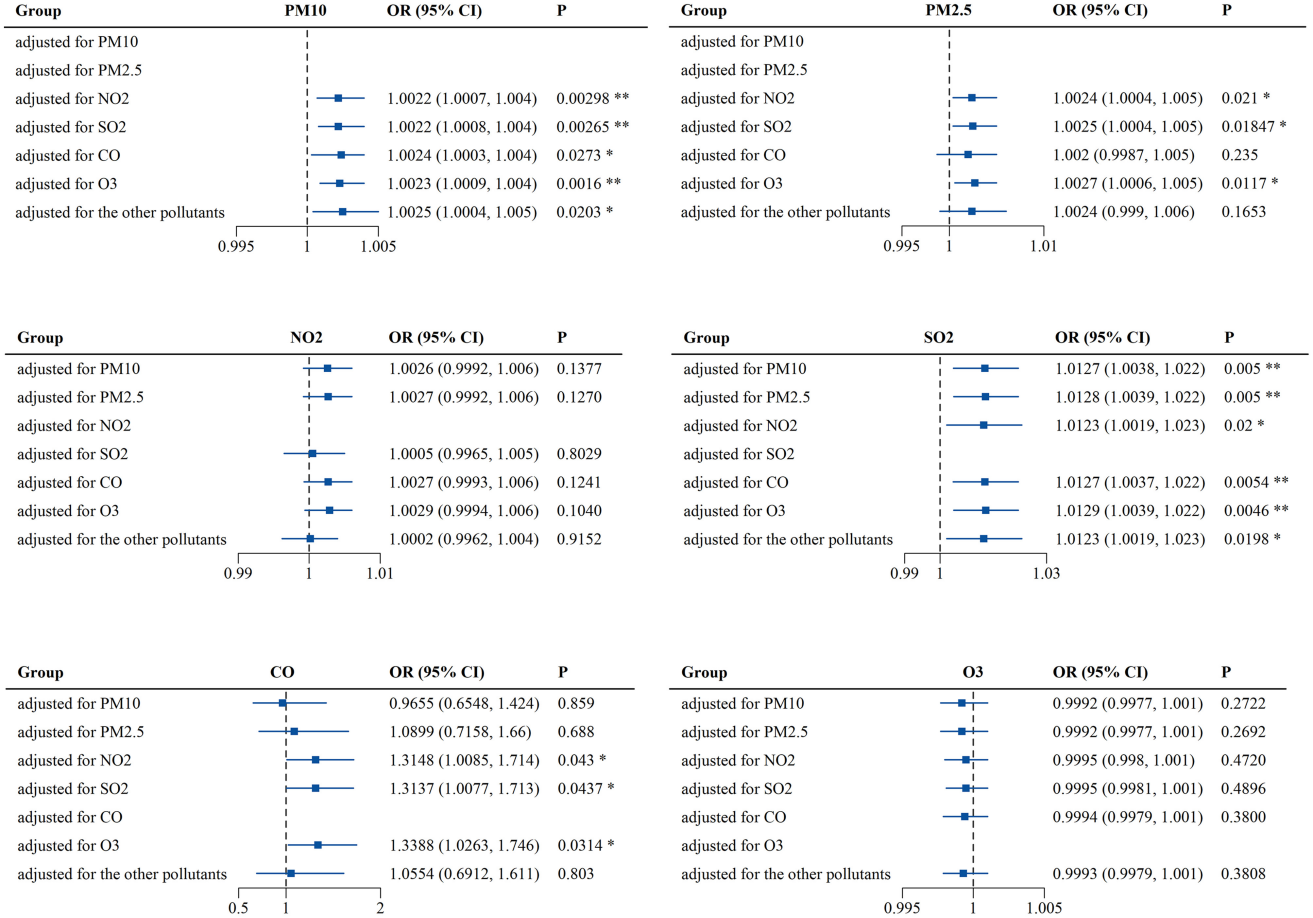
Associations of short-term exposure to air pollutants with outpatient visits for PALDO: multi-pollutant model. Conditional logistic regression was performed using a Cox proportional hazards regression model to calculate the odds ratios (OR) and 95% confidence interval (CI). Each panel shows the association between PM_10_, PM_2.5_, NO_2_, SO_2_, CO, and O_3_ and PALDO cases after adjustment for other pollutants. *P < 0.05, **P < 0.01. PM_10_, particulate matter <10 μm in diameter; PM_2.5_, fine particulate matter <2.5 μm in diameter; SO_2_, sulfur dioxide; NO_2_, nitrogen dioxide; CO, carbon monoxide; O_3_, ozone.

As the interactions between air pollutants were unavoidable, the multi-pollutant model was also used for analysis. In this model, the concentrations of air pollutants in the best lags and meteorological factors were chosen to be used as variables and controls, respectively. As previously mentioned, PM_2.5_ was strongly correlated with PM_10_ (*r* = 0.94) and its OR for PALDO outpatient visits was higher than that of PM_10_, so only PM_2.5_ was selected in the multi-pollutant model. Both PM_10_ and SO_2_ were significantly associated with PALDO outpatient visits, after adjusting for the other pollutants. Positive associations were also observed between PALDO outpatient visits and the pollutants PM_2.5_ and CO, after adjusting for a certain pollutant (Figure 2 and Supplementary Table S3).

**Figure 3:**
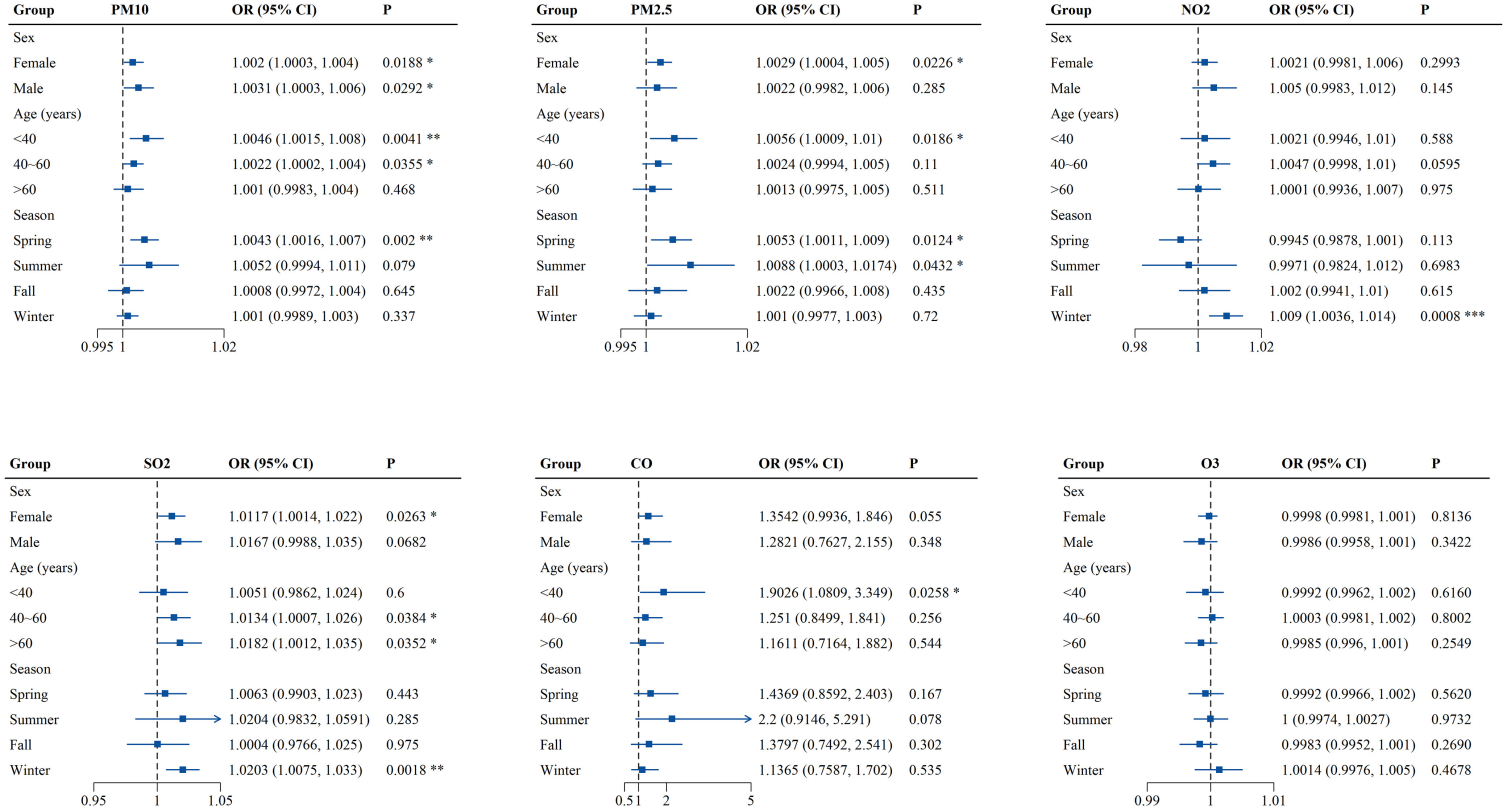
Associations of short-term exposure to air pollutants with outpatient visits for PALDO stratified by sex, age and season. Single-pollutant model was applied to calculate the odds ratios (OR) and 95% confidence interval (CI) in conditional logistic regression. Each panel shows sex-, age- and season-specific associations between PM_10_, PM_2.5_, NO_2_, SO_2_, CO, and O_3_ and PALDO cases. *P < 0.05, **P < 0.01, ***P < 0.001. PM_10_, particulate matter <10 μm in diameter; PM_2.5_, fine particulate matter <2.5 μm in diameter; SO_2_, sulfur dioxide; NO_2_, nitrogen dioxide; CO, carbon monoxide; O_3_, ozone.

Finally, sex-, age- and season-specific associations between air pollutants and outpatient visits for PALDO were analyzed. As shown in Figure 3 and Supplementary Table S4, PM_10_ was significantly associated with PALDO outpatient visits for both genders, while the associations between the pollutants (PM_2.5_ and SO_2_) and PALDO outpatient visits were only significant among females. In addition, the associations were significant for PM_10_, PM_2.5_, and CO in patients with age under 40, for PM_10_ and SO_2_ in patients with age between 40 and 60, and for SO_2_ in patients with age over 60. The season-specific results showed significant associations for PM_10_ and PM_2.5_ in spring, as well as NO_2_ and SO_2_ in winter. Besides, PM_2.5_ was significantly associated with outpatient visits for PALDO in summer. The *p*-values for the interaction between air pollutants and sex, age and season were shown in Supplementary Table S5. There was evidence of effect modification by age groups for PM_10_ (*p* = 0.045) and by season groups for NO_2_ (*p* = 0.001).

## Discussion

In the present study, we found that short-term exposure to higher levels of PM_10_, PM_2.5_, NO_2_, SO_2_, and CO was associated with increased risk of outpatient visits for PALDO in Hangzhou, China. Moreover, associations of PALDO outpatient visits with the pollutants (PM_10_, PM_2.5_, SO_2_, and CO) remained significant after adjusting for the certain pollutant in the multi-pollutant model. The subgroup differences associated with air pollutants varied by sex, age and season. To our knowledge, this is the first epidemiological study with a time-stratified case-crossover design to evaluate the risk of air pollution on outpatient visits for PALDO.

To date, several environmental risk factors have been reported to influence or contribute to the pathogenesis of PALDO. Tobacco smoke, one of the most common indoor pollutants, has been suspected as an etiological factor. Firstly, smoking has long been associated with dacryoliths, which are concretions formed in LDS. Early in 1965, Jones reported that the majority cases of dacryoliths were moderate or heavy smokers (36). Recent studies also have found a higher prevalence of smoking in patients with dacryoliths (37). The dacryoliths are believed to induce partial or complete obstruction of LDS, resulting in symptoms including epiphora, and acute dacryocystitis (38, 39). However, a large community based case-control study has refuted this claim by showing less prevalence of smoking among PALDO patients (40). Secondly, tobacco smoke can stimulate ocular surface and airways inflammation through an increase in inflammatory cells and the release of pro-inflammatory cytokines (41, 42). Pathological studies revealed that descending inflammation from the eye or ascending inflammation from the nose would contribute to the pathological changes of PALDO, yet severity of inflammation was not significantly different between smokers and nonsmokers with PALDO (43). Swimming pool exposure has been considered as another risk factor, as it was found to be independently associated with the development of PALDO (44). The possible explanation was chlorine, widely used to disinfect pool water, could react with ammonia in the water to form chloramines. The chloramines can cause significant eye and airways irritant symptoms, through the proposed mechanisms including inflammation, oxidative stress and hyperpermeability (45, 46). Since LDS is adjacent to the eye and nose, it would not be surprising to speculate the chloramines could also have similar effects on the luminal mucosa, which may contribute to PALDO pathogenesis. However, the pathogenic mechanism of these environmental factors on PALDO has not been clarified yet.

In this study, we evaluated the effects of six criteria air pollutants (PM_10_, PM_2.5_, NO_2_, SO_2_ CO, and O_3_) on outpatient visits for PALDO and found that short-term exposure to these air pollutants except O_3_ increased the possibility of outpatient visits. As a primary irritant, O_3_ can be hazardous to the eye, airways and skin owing to its oxidizing property (47). However, our results showed no significant association between O_3_ and PALDO outpatient visits in either single-pollutant or multi-pollutant model. The possible explanation is that the deleterious effects of O_3_ could take longer to present, and the relatively short analysis period may conceal the actual effect of O_3_ on the development of PALDO (48). In the subgroup analyses, female and young and middle-aged (< 40 and 40–60 years old) patients with PALDO were sensitive to more air pollutants. This gender difference may be related to the longer and narrowed bony nasolacrimal duct and lower levels of hormonal receptor expression in females, making them more susceptible to environmental risk factors such as air pollution (49, 50). Moreover, there are several plausible explanations for the age predilection observed in the estimates of pollutants effect by age. It is well known that young and middle-aged people are main labor force in the society, meaning that they spend more time outdoors for work and activity, so they may have greater exposure to air pollutants. On the other hand, air pollution may trigger a greater immune inflammatory response in these people due to their relatively active immune system than old people (51). Another interesting finding was that most air pollutants (PM_10_, PM_2.5_, NO_2_, and SO_2_) were significantly associated with PALDO outpatient visits in spring or winter. The differences are likely induced by seasonal differences in concentrations of air pollutants, as well as compromise of the mucosal barrier function in cold season (52). Further studies are needed to determine the dose-dependent effects of air pollutants on the pathogenesis of PALDO.

Multiple biological mechanisms could be involved in the association between air pollution exposure and the exacerbation of PALDO. First, air pollutants may damage the luminal mucosa of LDS. Several experimental studies found that PM_2.5_ led to cytotoxicity in human corneal epithelial cells, as well as goblet-cell hyperplasia in human conjunctiva (53, 54). In the nasolacrimal ducts from PALDO samples, ultrastructural changes were seen and characterized by focal areas of mucosal epithelial loss and basal layer hyperplasia (55). Second, inflammatory processes induced by air pollutants could be another explanation for this association. Previous studies have shown that air pollutants could stimulate both innate and adaptive immune responses, resulting in pro-inflammatory cytokines release, innate immune cells activation and B and T lymphocytes infiltration, which were also observed in the lacrimal sacs obtained from PALDO patients (56, 57). Third, air pollutants could indirectly influence the outpatient visits for PALDO with systemic comorbidities, such as hormonal imbalance, autonomic dysregulation and host defenses derangement (58–60).

It is noted that this study also has several limitations. Firstly, outpatient data from other clinics or hospitals in the city were not included, although our eye center attracted a large number of patients with eye diseases (about 1 million outpatient visits per year). Secondly, the city-level air pollution estimates may not represent the true exposures of patients, as the spatial-temporal variations of individual activities could not be measured. Thirdly, possible effect modifications by some risk factors like smoking and occupations were not evaluated due to limited patient information. Fourthly, we could not adjust for other potential confounders including holidays and the prevalence of Novel Coronavirus Disease 2019 (COVID-19). These confounding factors may affect the occurrence of PALDO to some extent.

## Conclusions

Our results showed positive associations between short-term exposure to air pollutants (PM_10_, PM_2.5_, NO_2_, SO_2_, and CO) and outpatient visits for PALDO in Hangzhou, China. Additionally, differences were also found in sex-, age- and season-specific associations. This study provides an insight into the role of air pollution in the pathogenesis of PALDO, and indicates the urgency to accelerate environmental protection and governance through policies and laws. Moreover, our findings can help the public to establish prevention and control measures for PALDO, including reducing air pollution exposure and maintaining a suitable environment.

## Data Availability

The original contributions presented in the study are included in the article/Supplementary material, further inquiries can be directed to the corresponding authors.
